# Early resuscitation of dengue shock syndrome in children with hyperosmolar sodium-lactate: a randomized single-blind clinical trial of efficacy and safety

**DOI:** 10.1186/s13054-014-0466-4

**Published:** 2014-09-05

**Authors:** Dadang H Somasetia, Tatty E Setiati, Azhali M Sjahrodji, Ponpon S Idjradinata, Djatnika Setiabudi, Hubert Roth, Carole Ichai, Eric Fontaine, Xavier M Leverve

**Affiliations:** Department of Pediatrics, Padjadjaran University, Bandung, Indonesia; Department of Pediatrics, Diponegoro University, Semarang, Indonesia; LBFA - INSERM U1055, Joseph-Fourier University, BP 53 38041 Grenoble, Cedex France; Service de Réanimation, Nice Sophia-Antipolis University, Nice, France

## Abstract

**Introduction:**

Dengue shock syndrome (DSS) fluid resuscitation by following the World Health Organization (WHO) guideline usually required large volumes of Ringer lactate (RL) that might induce secondary fluid overload. Our objective was to compare the effectiveness of the recommended volume of RL versus a smaller volume of a hypertonic sodium lactate solution (HSL) in children with DSS. The primary end point was to evaluate the effect of HSL on endothelial cell inflammation, assessed by soluble vascular cell adhesion molecule-1 (sVCAM-1) measurements. Secondarily, we considered the effectiveness of HSL in restoring hemodynamic fluid balance, acid–base status, and sodium and chloride balances, as well as in-hospital survival.

**Methods:**

A prospective randomized single-blind clinical trial including 50 DSS children was conducted in the Pediatrics Department of Hasan Sadikin Hospital, Bandung, Indonesia. Only pediatric patients (2 to 14 years old) fulfilling the WHO criteria for DSS and new to resuscitation treatments were eligible. Patients were resuscitated with either HSL (5 ml/kg/BW in 15 minutes followed by 1 ml/kg/BW/h for 12 hours), or RL (20 ml/kg/BW in 15 minutes followed by decreasing doses of 10, 7, 5, and 3 ml/kg BW/h for 12 hours).

**Results:**

In total, 50 patients were randomized and included in outcome and adverse-event analysis; 46 patients (8.2 ± 0.5 years; 24.9 ± 1.9 kg; mean ± SEM) completed the protocol and were fully analyzed (24 and 22 subjects in the HSL and RL groups, respectively). Baseline (prebolus) data were similar in both groups. Hemodynamic recovery, plasma expansion, clinical outcome, and survival rate were not significantly different in the two groups, whereas fluid accumulation was one third lower in the HSL than in the RL group. Moreover, HSL was responsible for a partial recovery from endothelial dysfunction, as indicated by the significant decrease in sVCAM-1.

**Conclusion:**

Similar hemodynamic shock recovery and plasma expansion were achieved in both groups despite much lower fluid intake and fluid accumulation in the HSL group.

**Trial Registration:**

ClinicalTrials.gov NCT00966628. Registered 26 August 2009.

## Introduction

Dengue is the most frequent mosquito-borne viral infection among human beings, with more than 50 million new infections being projected annually [1]. Although it resolves spontaneously in most cases, dengue hemorrhagic fever (DHF) and dengue shock syndrome (DSS) are among the leading causes of pediatric hospitalization. Mortality rates from 1% to 5% are frequently reported for DHF/DSS from centers experienced in fluid resuscitation, but rates up to 44% have occasionally been reported in cases of established shock [2].

The major pathophysiologic abnormality responsible for DHF/DSS is an acute increase in vascular permeability, leading to plasma leakage from the vascular to the extravascular compartment [2-5], which results in hypovolemia (biologically characterized by an hemoconcentration), responsible for a moderate to severe shock. It has been suggested that the dengue virus induces a swelling of endothelial cells [3], which may damage the tight-junction complexes, thus increasing vascular permeability. Usually, the capillary leakage resolves spontaneously by the sixth day of illness and is rapidly followed by full recovery. Microvascular permeability is intrinsically higher among children than in adults, which may explain why children are more prone than adults to DSS [4,6].

Endothelial cell inflammation is responsible for a high expression of VCAM-1 at the cell surface, leading to an increase in circulating sVCAM-1. It has been proposed that circulating sVCAM-1 levels may reflect the severity of the disease [7,8]. Therefore, from a theoretic point of view, the optimal treatment of DSS should address both the cause (endothelial dysfunction) and its consequence (hypovolemia).

Three controlled randomized double-blind trials comparing different resuscitation fluids in children with DHF/DSS failed to demonstrate any benefit of a particular fluid [9-11]. Comparing four fluids (Ringer lactate, NaCl 0.9%, dextran 70, and 3% gelatin), Dung *et al.* [9] did not find any difference in terms of administered volume and shock resolution. The same result was obtained in a comparable study with a larger population [10]. However, it was noted that the early administration of colloids to the most severely affected subgroup of patients was beneficial [10]. A randomized double-blind trial comparing three fluids (Ringer lactate, 6% dextran 70, 6% hydroxyethyl starch) for initial resuscitation of children with DSS established that isotonic crystalloids (RL) were as effective as colloids for initial resuscitation of children with moderate shock [11]. Consequently, the WHO recommends that patients with DSS should benefit from an immediate volume replacement with isotonic crystalloid solutions, followed by the use of plasma or colloid solutions for profound or continuing shock [2].

Totilac is a hypertonic lactate-based solution (see Table 1). It has been shown to be able to restore hemodynamic status and improve cardiac performance [12,13]. Moreover, as compared with other hypertonic crystalloid-based solutions, lactate can be used as an energy substrate by various tissues [14]. Finally, the hypertonic lactate-based solution has been shown to be effective in treating intracranial hypertension after traumatic brain injury, with a significantly more-pronounced effect than that of an equivalent osmotic load of mannitol, suggesting that sodium lactate may have an antiedema effect *per se* [15].

**Table 1:** **Composition of Ringer lactate (RL) and the hypertonic sodium lactate (HSL) solution Totilac**

	**Hypertonic sodium lactate**		**Ringer lactate**	
	**(HSL)**		**(RL)**	
**Content**	**m** ***M***	**g/L**	**m** ***M***	**g/L**
Na^+^	504.15	11.50	130.5	2.98
K^+^	4.02	0.16	4.02	0.16
Ca^2+^	1.36	0.050	0.67	0.024
Cl^−^	6.74	0.24	109.90	3.90
Lactate^−^	504.15	44.92	28.00	2.49
Total osmolarity (mosm/L)	1,020.42		273	
Organic/inorganic osmolarity (mosm/L)	504.15/516.27		28/245	

Based on this double property (intravenous fluid and antiedema effect), the present prospective randomized study was designed to compare the efficacy and safety of Totilac with RL for resuscitation of DHF/DSS children. Our hypothesis was that sodium lactate could improve endothelial cell function and decrease capillary leakage, thus minimizing fluid overload.

## Methods

**Figure 1:**
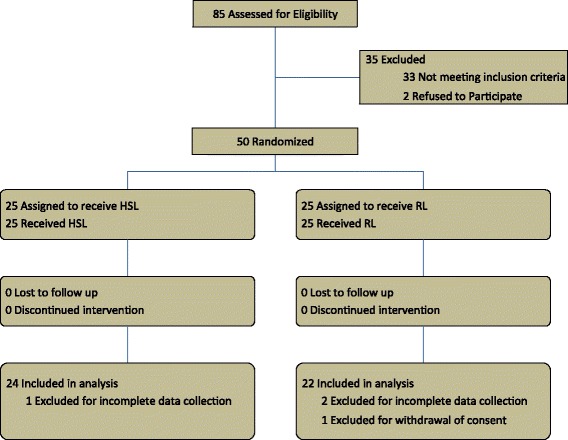
**Profile of the randomized controlled trial.**

Fifty children with severe DSS were enrolled in this prospective, randomized, single-blind study (see Figure 1), conducted in the Pediatrics Department of Hasan Sadikin Hospital, Bandung, Indonesia, between May 2008 and April 2009. Patients were randomly assigned to RL or hypertonic sodium lactate solution (HSL) groups. Randomization was performed independently by using a table of random numbers and balanced blocks of four, with sealed opaque envelopes sequentially numbered. The enrollment of patients was performed by the pediatrician, and the assignment to intervention, by the study coordinator.

The Ethics Committee of Hasan Sadikin Hospital had approved this protocol before the study. Informed consent had also been obtained from the patients’ relatives.

**Figure 2:**
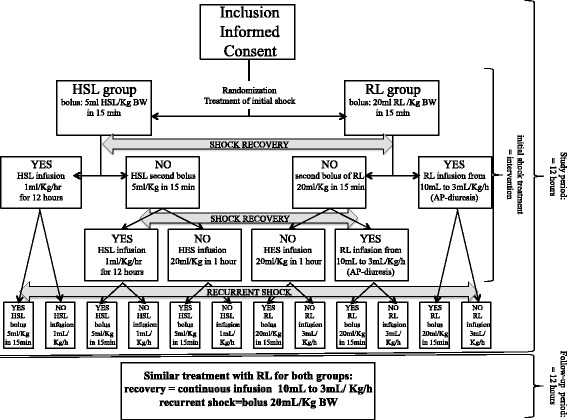
**Study flow chart.**

The intervention period ran for the first 12 hours of treatment and was followed by a 12-hour observation period (see Figure 2) extended to 48 hours for sVCAM-1 measurement. The outcome, complications, adverse events, and concomitant treatments were recorded throughout the entire hospital stay until discharge. Only pediatric patients (2 to 14 years old) fulfilling the WHO criteria for DSS and new to resuscitation treatment were eligible. Based on anamnesis, physical examination, and/or laboratory tests, patients with another viral or bacterial infection, severe renal failure (creatinine >180 μ*M* - because of the high amount of sodium infused), chronic diarrhea (because it may influence urine output), liver failure (SGOT and SGPT >20 times normal), severe malnutrition and diabetes mellitus (because they may change lactate metabolism), and hematologic disorders (because hemostasis was one studied parameter) were excluded.

### Shock management

According to the randomization, eligible patients received an initial bolus infusion of either HSL (5 ml/kg BW) or RL (20 ml/kg BW) for 15 minutes (Figure 2). If they did not recover from the shock rapidly with the first bolus, a second loading infusion with the same solution at the same dosage was infused once again. If the second treatment failed with a persistent shock, patients received hydroxyl-ethyl starch (HES, 130/0.40) infusion (20 ml/kg BW in 15 to 30 minutes) with a maximum dose of 50 ml/kg BW in 24 hours. Note that the WHO recommends colloid solutions for profound or continuing shocks, whereas HES administration was in routine practice, and concern about its safety was not reported before this study started.

If the shock was reversed, patients received a maintenance dose of the studied solution for 12 hours: 1 ml/kg BW/h of HSL, or decreasing doses of 10, 7, 5, and 3 ml/kg BW/h of RL, based on patients' hemodynamic conditions (heart rate, systolic blood pressure, urine output), which corresponds to the standard protocol of DSS management in Hasan Sadikin Hospital according to the WHO recommendations. If the shock recurred within the first 12 hours, the studied solution was infused once again, as in the initial shock management. After the 12^th^ hour (that is, during the follow-up period), both groups received 3 ml/kg BW/h RL according to the WHO recommendations. If the shock recurred during the follow-up period, the standard protocol of DSS management according to the WHO recommendations was applied (RL infusion with HES administration if necessary).

### Studied parameters

As markers of hemodynamic status and tissue oxygenation, blood pressure, heart and respiratory rates, Glasgow Coma Scale score (GCS), body temperature, and urine output were monitored before (T0) and after 15 and then 30 minutes, every hour until the hour 6, and then 9, 12, 18, and 24 hours after fluid administration.

Fluid infusion and output were measured and documented. Laboratory blood/plasma parameters including hemoglobin, hematocrit, thrombocyte and leukocyte counts, electrolytes (sodium, potassium, chloride), plasma osmolality, lactate, glucose, albumin, total protein concentrations, SGOT, SGPT, creatinine, venous gas analysis, prothrombin (PT), activated partial thromboplastin time (aPTT), fibrinogen levels, and D-dimer were determined after 0, 6, 12, and 24 hours. sVCAM-1 values were measured after 0, 24, and 48 hours with the ELISA method (R&D Systems, Human sVCAM-1/CD106 Quantikine ELISA Kit, catalog number DVC00). Any adverse events or serious adverse events that occurred during the study period were recorded and reported for all included patients. All concomitant medications and their complement, including dosages, were also documented.

### Study end points

The primary end point was the effectiveness of HSL to decrease endothelial cell inflammation assessed by sVCAM-1 measurements during the first 48 hours of DSS. Secondary end points considered the effectiveness of HSL to restore hemodynamic and tissue-oxygenation parameters, fluid balance, acid–base status, sodium and chloride balances, as well as in-hospital survival.

### Sample-size determination and statistical analysis

G*Power3.1 [16] was used to calculate the sample size for a five-repeated-measure ANOVA with an alpha error of 0.05, a power of 80%, a correlation among repeated measures of 0.3, and an effect size of 0.3. The theoretical sample size was *n* = 21 in each group. We chose to include 25 subjects per group.

Unless otherwise indicated, data are expressed as mean ± SEM. Qualitative variables were analyzed by using the Fisher Exact test (Table 2). The effect of DSS treatment over time was analyzed with ANOVA for repeated measures, indicating the significance of the different treatments (treatment effect) and evolution (time effect) (Table 3). Data exhibiting non-normal distribution were analyzed with nonparametric unpaired (Mann-Whitney) or paired (Wilcoxon) tests, as indicated in the legends.

**Table 2:** **Baseline quantitative data (mean (SEM))**

	**HSL**	**RL**
	***n*** **= 24**	***n*** **= 22**
Age, years	8.7 (0.6)	7.6 (0.7)
Body weight, kg	27.1 (3.0)	22.5 (2.0)
Height, cm	126.7 (4.4)	118.1 (3.8)
Sex ratio (F:M)	11:13	11:11
GCS	14.4 (0.3)	14.8 (0.2)
Systolic pressure, mm Hg (*n* = 9 + 9)	89.4 (4.7)	86.1 (3.7)
Diastolic pressure, mm Hg (*n* = 9 + 9)	61.1 (6.5)	65.6 (4.4)
Heart rate, beats/min	133.9 (4.8)	121.7 (3.7)
Respiratory rate, breaths/min	34.4 (2.2)	33.4 (1.7)
Temperature, °C	36.7 (0.2)	36.2 (0.2)
DHF grade III	9	9
DHF grade IV	15	13
Hematocrit, %	41.9 (0.93)	42.4 (1.2)
Hemoglobin, g/dl	14.5 (0.3)	14.7 (0.5)
Leukocyte, mm^−3^	6,217 (854)	6,000 (755)
Thrombocyte, mm^−3^	47,042 (7,693)	59,409 (7,421)
pH (venous)	7.40 (0.03)	7.37 (0.02)
pCO_2_ (venous), mm Hg	24.9 (1.5)	24.8 (1.4)
pO_2_ (venous), mm Hg,	55.6 (7.2)	66.9 (7.8)
CO_3_H^−^ (venous), m*M*	15.2 (0.9)	13.8 (0.7)
BE (venous), m*M*	−7.6 (1.1)	−10.3 (0.9)
Na, m*M*	129.8 (1.2)	128.1 (0.9)
K, m*M*	4.36 (0.14)	4.64 (0.17)
Cl, m*M*	94.3 (1.3)	95.8 (1.1)
Osmolality, mOsm/kg H_2_O	284.0 (4.6)	275.4 (1.8)
Glucose, m*M*	6.74 (0.37)	6.11 (0.43)
Lactate, m*M*	4.71 (0.67)	3.81 (0.85)
Creatinine, mg/dl	0.68 (0.06)	0.55 (0.04)
Albumin, g/L	32.8 (1.6)	34.5 (1.4)
Protein, g/L	59.3 (2.4)	62.2 (2.9)
SGOT μu.L^−1^	798 (422)	387 (106)
SGPT u/L	283 (158)	119 (28)
PT, seconds	12.0 (0.7)	11.5 (0.7)
aPTT, seconds	42.0 (4.5)	36.6 (2.4)
D-dimer ng/ml	627 (198)	455 (85)
Fibrinogen, mg/L	163.7 (14.6)	154.3 (16.7)

**Table 3:** **Randomization, treatment, and evolution of DSS: comparison between HSL and RL**

**Patients included**	**50**		
**Randomization**	**HSL**	**RL**	***P*** *
Patients randomized	25	25	
Patients withdrawn	1	3	0.60
Patients studied	24	22	
Per protocol analysis (*n* = 46)			
Initial shock treatment (number of patients)			
One bolus/two boluses	18/6	17/5	>0.99
HES infusions	4	1	0.30
Number of recurrent shocks within the first 12 hours (number of patients)			
Yes/No	7/17	2/20	0.14
HES infusions	1	0	0.48
Total HES infusions (24 hours)	10	1	0.005
Concomitant therapies			
Blood products			
Thrombocytes	1	4	0.18
Cryoprecipitate	0	2	0.22
Fresh frozen plasma	1	3	0.34
Antibiotics	7	4	0.60
Furosemide	3	3	>0.99
Catecholamines	1	1	>0.99
Antipyretic	6	1	0.13
Total colloid (HES + plasma) (24 hours)	11	4	0.06
Intent-to-treat analysis (*n* = 50)			
Outcome			
Recovery	22	20	0.70
Forced discharge	2	2	>0.99
Death	1	3	0.60
Adverse events/complications			
DIC	3	5	0.70
Encephalopathy	4	3	>0.99
Respiratory distress syndrome	1	0	>0.99
Acute liver failure	0	2	0.47

*Fisher Exact test.

## Results

### Studied population

The 50 enrolled and randomized patients were included in outcome and adverse-event analysis (intent-to-treat analysis). Forty-six patients completed the protocol and were fully analyzed (24 and 22 subjects in the HSL and RL groups, respectively) whereas four patients were excluded: three for incomplete data collection and one for withdrawal of consent. As shown in Table 2, because of randomization, the two groups were not different before treatment in terms of nutritional status, severity of disease, or baseline parameters.

**Table 4:** **Evolution of blood parameters at 0, 6, 12, and 24 hours after initiation of shock treatment expressed as mean (SEM)**

	**Treatment**	**T0**	**T6**	**T12**	**T24**	***P*** **treatment**	***P*** **time**	***P i*** **nteraction**
Leukocytes, mm^−3^	HSL	6,217 (854)	5,600 (566)	6,665 (698)	7,100 (764)	0.862	0.001	0.844
	RL	6,000 (755)	5,805 (842)	6,623 (816)	7,864 (1,016)			
Thrombocytes, mm^−3^	HSL	47,042 (7,693)	44,583 (6,102)	40,348 (6,001)	48,773 (5,817)	0.297	0.016	0.529
	RL	59,409 (7,421)	48,727 (5,870)	50,455 (6,583)	55,909 (4,867)			
pH (venous)	HSL	7.40 (0.03)	7.50 (0.01)	7.50 (0.02)	7.46 (0.01)	<0.0001	<0.0001	0.100
	RL	7.37 (0.02)	7.40 (0.01)	7.41 (0.01)	7.39 (0.01)			
CO_3_H^−^ (venous), m*M*	HSL	15.20 (0.87)	21.56 (1.04)	26.53 (1.19)	25.18 (0.73)	<0.0001	<0.0001	<0.0001
	RL	13.76 (0.72)	15.21 (0.86)	16.94 (0.72)	19.35 (0.85)			
Na, m*M*	HSL	129.8 (1.2)	129.8 (1.2)	130.0 (1.3)	130.6 (1.3)	0.935	0.013	0.081
	RL	128.1 (0.9)	129.0 (1.0)	130.3 (0.8)	132.7 (1.3)			
K, m*M*	HSL	4.36 (0.14)	3.42 (0.13)	3.23 (0.11)	3.18 (0.11)	<0.0001	<0.0001	0.001
	RL	4.64 (0.17)	4.26 (0.10)	4.23 (0.16)	4.28 (0.15)			
Cl, m*M*	HSL	94.3 (1.3)	90.5 (1.2)	89.5 (1.5)	92.5 (1.1)	<0.0001	0.003	<0.0001
	RL	95.8 (1.1)	97.6 (1.0)	99.6 (1.1)	100.9 (0.9)			
Osmolality, mOsm/Kg H_2_O	HSL	284.0 (4.6)	277.9 (2.2)	278.5 (2.6)	276.4 (2.2)	0.240	0.416	0.104
	RL	275.4 (1.8)	274.2 (2.4)	275.2 (1.5)	279.5 (2.6)			
Glucose, mM	HSL	6.74 (0.37)	6.56 (0.27)	6.60 (0.39)	5.72 (0.35)	0.003	0.001	0.355
	RL	6.11 (0.43)	5.14 (0.27)	5.35 (0.33)	4.85 (0.29)			
Lactate, mM	HSL	4.71 (0.67)	2.94 (0.41)	2.93 (0.32)	1.65 (0.20)	0.313	<0.0001	0.183
	RL	3.81 (0.85)	1.87 (0.30)	2.11 (0.44)	2.24 (0.54)			
Creatinine, mg/dl	HSL	0.677 (0.063)	0.569 (0.066)	0.631 (0.088)	0.607 (0.105)	0.070	<0.0001	0.552
	RL	0.549 (0.039)	0.440 (0.023)	0.466 (0.030)	0.416 (0.023)			
Albumin, g/L	HSL	32.8 (1.6)	29.5 (1.1)	28.5 (1.1)	26.5 (0.9)	0.695	<0.0001	0.287
	RL	34.5 (1.4)	28.5 (0.8)	28.5 (0.7)	27.6 (0.9)			
Protein, g/L	HSL	59.3 (2.4)	50.7 (2.3)	48.5 (2.6)	48.0 (1.8)	0.264	<0.0001	0.990
	RL	62.2 (2.9)	53.1 (1.8)	51.3 (1.3)	50.2 (1.6)			
SGOT u/L	HSL	798 (422)	870 (383)	765 (373)	503 (191)	0.636	0.776	0.263
	RL	387 (106)	512 (229)	631 (363)	656 (355)			
SGPT u.L^−1^	HSL	283 (158)	258 (103)	245 (109)	185 (76)	0.678	0.934	0.267
	RL	118 (27)	181 (87)	218 (122)	229 (119)			
PT, seconds	HSL	12.0 (0.7)	12.2 (0.8)	12.2 (0.6)	11.4 (0.5)	0.443	0.221	0.094
	RL	11.5 (0.7)	11.4 (1.0)	13.6 (2.8)	17.8 (4.6)			
aPTT, seconds	HSL	42.0 (4.5)	49.4 (4.7)	50.6 (4.8)	39.9 (2.3)	0.179	0.386	0.117
	RL	36.6 (2.4)	39.9 (2.5)	38.3 (3.4)	44.6 (7.9)			
Fibrinogen, mg/L	HSL	163.7 (14.6)	123.1 (10.9)	131.7 (11.1)	124.1 (7.2)	0.443	<0.0001	0.734
	RL	154.2 (16.7)	117.6 (8.8)	110.5 (7.9)	118.4 (9.6)			

Repeated measures ANOVA between HSL and RL: *P treatment,* effect of treatment; *P time,* effect of time; *P interaction,* interaction between treatment and time.

### Initial shock treatment and outcome

As shown in Table 3, no statistical difference was found in the success rate of a single bolus infusion (*P* > 0.99) or in shock recurrence within the first 12 hours (*P* = 0.14). The needs of HES administration for initial shock treatment or shock recurrence within the first 12 hours were not different (*P* = 0.30 and *P* = 0.48 for initial and recurrent shock, respectively). However, HES infusion was more frequently used during the follow-up period (T12 to T24) in the HSL group (5 versus 0), leading to a significantly more frequent total HES administration (intervention and follow-up periods) in the HSL group (10 versus 1, *P* = 0.005).

No difference was noticed regarding the outcome. The full recovery rate was similar in the two groups (*P* = 0.70). Two patients in each group left the hospital on hospital discharge against medical advice after the intervention period. One versus three people died in the HSL and RL groups, respectively (*P* = 0.60). All four died because of irreversible/terminal shock and multiorgan failures with disseminated intravascular coagulation.

Concomitant therapies did not significantly differ between the two groups (Table 3). However, it could be underlined that, due to the occurrence of hematologic disorder, blood products (that is, thrombocytes, cryoprecipitate, and fresh frozen plasma) were required for 0ne and five patients in the HSL and RL groups, respectively (*P* = 0.09, Fisher Exact test), corresponding to two and nine blood products in the HSL and RL groups, respectively. Note that the frequency of use of colloids (HES + fresh frozen plasma) was not significantly different in the two groups.

### Comparison of the two fluid regimens on clinical parameters, intravascular volume expansion, and fluid balance

**Figure 3:**
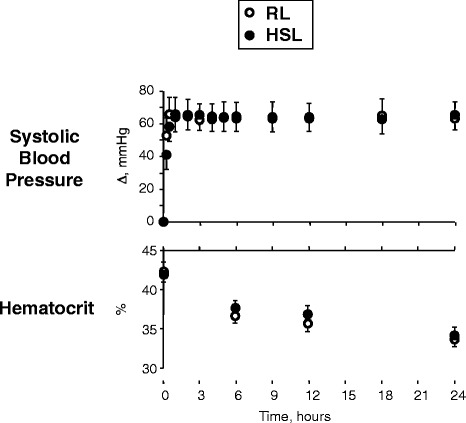
**Effect of treatments on systolic blood pressure and hematocrit.** Open circles, RL; solid circles, HSL.

As shown in Figure 3, systolic blood pressure had improved very significantly at the end of the bolus infusion (*P* < 0.0001) in both groups. This improvement persisted through the intervention and follow-up periods in both groups, with no difference between the two groups (*P* = 0.90). Hematocrit decreased significantly (*P* < 0.0001) in both groups, with no interaction between time and groups. Note that no patient received red cell packs. The urinary output rate (not shown) increased immediately after the beginning of fluid administration (*P* < 0.0001), with a more-pronounced effect in the RL group (*P* = 0.007).

**Figure 4:**
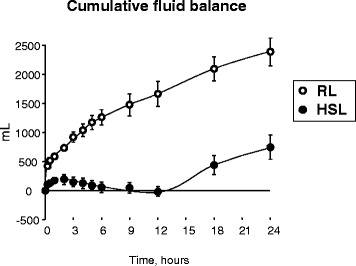
**Effect of treatments on fluid balance.** Open circles, RL; solid circles, HSL.

According to the protocol, patients in the RL group received much more fluid than those in the HSL group. The difference increased with time during the intervention period, reaching a four- to fivefold higher intake in the RL group than in the HSL group after 12 hours (115.9 ± 3.8 versus 26.2 ± 2.9 ml/kg/12 h, for RL and HSL, respectively, *P* < 0.0001). During the follow-up period, changes in fluid intake were parallel, as both groups then received the same treatment. Cumulative urine output was slightly but significantly (*P* = 0.018) higher in the RL group. However, as shown in Figure 4, the difference in fluid balance between the two groups was significant (*P* < 0.0001). The RL group accumulated 107 ± 7 ml/kg/24 h, whereas the HSL group accumulated only 35 ± 10 ml/kg/24 h. Note that Figure 4 takes into account all the fluids infused (crystalloids, HES, and concomitant therapies, including fresh frozen plasma). Interestingly, at the end of the intervention period (H12), when the initial shock had already been successfully treated for a couple of hours in the two groups, the fluid balance did not differ from zero in the HSL group (−0.2 ± 0.2 ml/kg/12 h; *P* = 0.95), whereas it was positive in the RL group (+75.9 ± 6.0 ml/kg/12 h; *P* < 0.0001). In other words, the fluid balance became positive in the HSL group only after these patients had been shifted from HSL to RL.

### Comparison of the two fluid regimens on biologic parameters

The main biologic parameters are shown in Table 4, before intervention, at hours 6, 12 (end of intervention), and 24 (end of follow-up period). Venous blood pH increased with both treatments during the intervention period, with a higher increase in the HSL than in the RL group. Increase in bicarbonate also was greater in the HSL group. No difference between the two treatments was found on plasma sodium concentration. Plasma potassium concentration decreased over time in both groups, with a larger decrease in the HSL group. Interestingly, plasma chloride concentration significantly decreased between T0 and T12 in the HSL group and then increased during the follow-up period, whereas it continuously increased in the RL group. Although the amount of infused lactate was quite substantial in the HSL group, blood lactate concentration did not increase in either the HSL or the RL group. We found no difference between the two groups for plasma osmolality, blood glucose concentration, creatinine, albumin, total protein, or transaminases.

**Figure 5:**
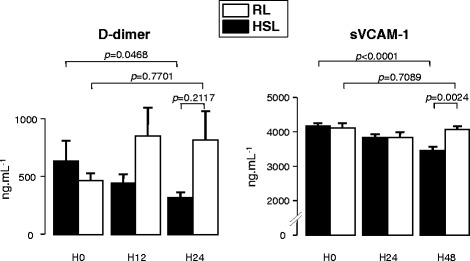
**Effect of treatments on homeostasis (D-dimer) and endothelial cell dysfunction (sVCAM-1).** Open bars, RL; solid bars, HSL. Comparisons were performed by using Wilcoxon tests for paired data (effect of time) and Mann–Whitney tests for unpaired data (difference between the two groups).

As regards hemostasis, the treatments did not affect PT or aPTT, while they both brought down fibrinogen. As seen in Figure 5, D-dimer did not significantly change (*P* = 0.7701) after 24 hours in the RL group, whereas it decreased in the HSL group (*P* = 0.0468), the difference between the two groups at the 24^th^ hour remaining insignificant, however (*P* = 0.2117). sVCAM-1 did not change in the RL group after 48 hours (*P* = 0.7089), whereas it significantly and continuously decreased in the HSL group (*P* = 0.0013 between T0 and T24, *P* = 0.0116 between T24 and T48, *P* < 0.0001 between T0 and T48), with a significant difference between the two groups at the 48^th^ hour (*P* = 0.0024).

### Comparison of the two fluid regimens on sodium and chloride balance

**Figure 6:**
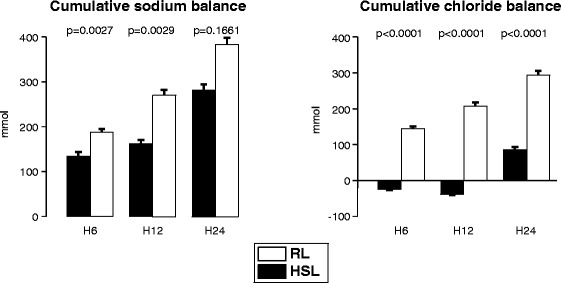
**Effect of treatments on sodium and chloride balance.** Open bars, RL; solid bars, HSL. Comparisons were performed by using Mann–Whitney tests.

As seen in Figure 6, cumulative sodium load was positive in both groups at any time and significantly higher in the RL than in the HSL group at T6 (*P* = 0.0027) and T12 (*P* = 0.0029). Indeed, despite a lower sodium concentration in RL than in HSL (Table 1), the more-substantial fluid infusion required led to a superior sodium load in the RL group.

Chloride cumulative balance significantly increased during the intervention (*P* = 0.0006) and the follow-up period (*P* = 0.0019) in the RL group. On the contrary, it significantly decreased (*P* = 0.0051) during the intervention period in the HSL group. During the follow-up period (that is, when RL was infused in both groups), the chloride cumulative balance became positive in the HSL group (*P* < 0.0001), but remained significantly lower than in the RL group (*P* < 0.0001).

## Discussion

It is well acknowledged that the dengue virus can lead to endothelial cell dysfunction responsible for capillary leakage with fluid extravasation from intravascular bed toward interstitial space. This leads to a severe hypovolemic shock in the absence of relevant fluid loss such as vomiting or diarrhea. In the present study, the recovery of such a severe hypovolemic shock without (first 12 hours) or with minimal (24 hours) net expansion of body fluids in the HSL group (see Figure 4) strongly suggests that HSL treatment favored a decrease of capillary leakage, allowing interstitial fluid to reintegrate the vascular bed.

To the best of our knowledge, all the treatments applied to DSS are symptomatic as regards shock or hemorrhagic disorder correction, and etiopathogenic treatments aiming at limiting the inflammatory response or virus invasion are reputed to be unsuccessful. The fact that sVCAM-1 decreased in patients receiving HSL suggests that this treatment might have improved the endothelial cell function.

Quite obviously, the plasma-expander effect of HSL might be related to its hyperosmolarity because a similar effect has been reported with hypertonic saline [17-19]. However, although HSL is hyperosmolar (see Table 1), it is less hypertonic than a sodium-chloride solution with the same osmolarity, because the monocarboxylate carrier allows lactate to cross the cellular plasma membrane [20]. In other words, lactate is expected to leave the intravascular bed to spread inside the cells. In agreement with such a property, lactate concentration did not increase in the HSL group.

Once into the cell, lactate is rapidly metabolized (mainly in glucose or in CO_2_ plus H_2_O), whereas Na^+^ is eliminated in urine. However, to maintain urine electroneutrality, negative charges must be eliminated with Na^+^, including Cl^−^, which results in a net negative chloride balance (see Figure 6). Because chloride loss was dramatically higher than the decrease in plasma chloride concentration (compare Figure 6 with Table 4), the net negative chloride balance was done mostly at the expense of intracellular chloride. Recent data on Na^+^, K^+^, and Cl^−^ co-exchange support a significant role of chloride balance on cell-volume regulation [21-23]. Hypothesizing that the dengue virus leads to endothelial cells swelling that in turn increases endothelium permeability because of changes in the cell shape, we propose that lactate might return the cell volume back to normal, thus correcting capillary leakage. Note that a negative chloride balance and a decrease in cerebral edema have also been reported in traumatic brain-injured patients infused with sodium lactate [15], whereas HSL has been shown to reduce the number of intracranial hypertensive episodes after severe traumatic brain injury [24].

From these considerations, it appears that HSL may affect intra- versus extracellular fluid distribution in at least two different ways: sodium-related hypertonicity and electrogenic imbalance corrected by chloride efflux from intracellular space.

There are several limitations to this study. The rate of lactate infusion used in this work was based on previous data indicating an average endogenous rate of lactate turnover of 10 to 14 mmol/kg/24 h and plasma clearance of about 10 to 14 ml/kg [25,26], as well as on previous administration of lactate to patients [12,15]. Note that the initial bolus infusion of HSL (5 ml/kg BW) was as effective as the initial bolus infusion of RL (20 ml/kg BW) for the acute shock management, as indicated by a similar initial recovery, including the success rate of a single bolus infusion.

However, the number of patients requiring HES for the initial shock treatment was higher (although not significantly different) in the HSL group. The fluid infusion rate of HSL after boluses (1 ml/kg/h) or the duration of the study period (during the first 12 hours) may have been underestimated for some patients. Indeed, recurrent shocks were more frequent (although not significantly so) in the HSL group during the study period (first 12 hours). Moreover, HES administration was required during the follow-up period in the HSL group (that is, once sodium lactate infusion had been stopped). This may suggest that a higher rate of fluid infusion after boluses (for instance, 2 ml/kg/h) is required, or that the study period should be extended for some patients. Note, however, that blood products were more frequently used in the RL group, which accounts for a significant volume of perfusion and may play down the difference in HES use between the two groups. Indeed, considering the perfusion of colloids (HES and fresh frozen plasma), no significant difference was noted between the two groups.

The outcome was similar in the two groups, as were the complications and the absence of any treatment-related side effect. We noted a slightly higher mortality in the RL group as compared with the HSL group, but this difference was not significant. Indeed, because this study was not powered to evaluate a beneficial outcome (but only the physiological consequences of the two treatments), a large multicentric study would be needed to do so.

## Conclusion

This randomized clinical trial has shown that the use of hyperosmolar sodium-lactate solutions in the Dengue shock syndrome allowed the treatment of such a hypovolemic shock with minimal fluid accumulation. Besides the fact that this treatment helped prevent fluid overload, the reduction of sVCAM may have contributed to the resolution of shock. Despite the preliminary nature of our data, the putative mechanism of action of hyperosmolar sodium-lactate solution is of sufficient interest to justify a large-scale clinical trial.

## Key messages

- Hyperosmolar sodium-lactate solutions allowed the correction of dengue shock syndrome with minimal fluid overload.
- This strongly suggests that hyperosmolar sodium-lactate favored a decrease of capillary leakage, allowing interstitial fluid to reintegrate the vascular bed.

## Abbreviations

aPTT: Activated partial thromboplastin time
DHF: Dengue hemorrhagic fever
DIC: disseminated intravascular coagulation
DSS: Dengue shock syndrome
GCS: Glasgow coma score
HES: hydroxyl-ethyl starch
HSL: hypertonic sodium lactate solution
PT: prothrombin time
RL: Ringer lactate
sVCAM-1: soluble vascular cell adhesion molecule-1
WHO: World Health Organization
